# Dataset on the performance of a photovoltaic solar water pump in coffee plantations using response surface methodology (RSM)

**DOI:** 10.1016/j.dib.2026.112467

**Published:** 2026-01-14

**Authors:** Nopparat Suriyachai, Torpong Kreetachat, Saksit Imman

**Affiliations:** School of Energy and Environment, University of Phayao Tambon Maeka, Amphur Muang, Phayao 56000, Thailand

**Keywords:** Solar irradiance, Panel inclination, Panel surface temperature, Experimental dataset, Pump efficiency, Response surface methodology, Renewable energy, Coffee cultivation

## Abstract

This dataset presents experimental data on the performance of a photovoltaic (PV) solar-powered water pumping system installed in a coffee plantation in Chiang Mai province, Thailand. The system performance was evaluated through controlled experiments using response surface methodology (RSM). Three independent variables were systematically varied: solar irradiance (300–900 W/m²), panel inclination (15–35°), and panel surface temperature (30–60°C). A total of 15 experimental runs were conducted, and the pumping efficiency (%) was recorded under each condition. Statistical analyses, including analysis of variance (ANOVA) and regression modeling, were applied to evaluate the effects of the individual variables and their interactions on system performance. The dataset includes raw and processed measurements, regression coefficients, and response surface parameters, enabling replication and further analysis. Perturbation plots, 3D surface plots, and contour plots provide detailed visualizations of the relationships between environmental factors and system efficiency. The optimal operating conditions were identified at a solar irradiance of 600 W/m², a panel inclination of 25°, and a panel surface temperature of 45°C, corresponding to a predicted maximum efficiency of 76.3–77.0%.

This dataset can be reused for designing optimized solar water pumping systems, validating predictive models, and comparing system performance under different environmental conditions or geographic locations. It also serves as a reference for researchers in renewable energy system optimization and agricultural water management. The data provide high-resolution, experimentally validated information on the combined effects of solar irradiance, panel inclination, and panel surface temperature on PV water pumping efficiency. Unlike previous studies, it includes detailed quantitative analysis specific to coffee-growing regions in Northern Thailand, along with regression models and visualizations that can guide both experimental replication and predictive modeling under similar climatic and agricultural conditions

Specifications Table

**Table:** 

Subject	Earth & Environmental Sciences
Specific subject area	Photovoltaic water pumping efficiency analysed via response surface methodology.
Type of data	Table, Graph, Figure, 3D surface plot, Contour plot; Raw, Processed, Analysed.
Data collection	Experimental data were collected from a PV solar-powered water pumping system installed in a coffee plantation in Chiang Mai, Thailand. Pumping efficiency (%) was measured under varying solar irradiance (300–900 W/m²), panel inclination (15–35°), and panel surface temperature (30–60°C). Response Surface Methodology (RSM) was applied for experimental design and analysis using Design-Expert software (v10).
Data source location	Coffee plantation, Chiang Mai Province, Thailand (Approx. 18.823854° N, 99.106530° E); Data stored at [School of Energy and Environment University of Phayao
Data accessibility	Repository name: Mendeley Data Data identification number: 10.17632/fgv56hzxnt.1 Direct URL to data: https://data.mendeley.com/datasets/fgv56hzxnt/1
Related research article	None.

## 1 Value of the Data

• These data provide field-measured performance of a photovoltaic (PV) solar-powered water pumping system under real environmental conditions in a coffee-growing area of Chiang Mai, Thailand.
• The dataset includes three key inputs solar irradiance, panel inclination, and panel surface temperature together with pumping efficiency, offering a complete set of variables relevant to PV system analysis.
• Researchers can reuse these data to develop or validate statistical models, optimization frameworks, or simulation tools for PV water pumping applications in agricultural environments.
• The inclusion of RSM model parameters, ANOVA outputs, and residual diagnostics allows other researchers to verify model structure, compare modelling approaches, or perform sensitivity and uncertainty analysis.
• These data are useful for researchers and engineers working on renewable energy, irrigation system design, or environmental performance assessment, particularly in regions with similar climatic conditions.

## 2 Background

This research investigates the operating characteristics of a photovoltaic (PV) solar-powered water pumping system installed in a coffee plantation in Chiang Mai province, Thailand. The dataset was generated to document how key environmental and system parameters: solar irradiance, panel inclination, and panel surface temperature vary under real field conditions and how they are incorporated into a structured performance analysis framework [1].

The methodological basis of the study follows response surface methodology (RSM), which requires a statistically designed experiment and systematic variation of independent variables to support quadratic model development [2]. Accordingly, the experimental design employed parameter ranges representative of typical highland agricultural conditions. The dataset includes residual analysis, analysis of variance (ANOVA), and regression coefficients to fully describe the statistical structure of the RSM model [3], enabling model reproducibility and transparent assessment of factor effects. Previous work by Waila et al. [4] has demonstrated the effectiveness of RSM for optimizing photovoltaic (PV) solar-powered water pumping systems. In contrast to existing PV water pumping datasets that mainly report long-term monitoring or steady-state performance, this dataset provides a structured experimental matrix based on response surface methodology, including controlled multi-level parameter variations and complete statistical outputs, enabling direct model reconstruction and improved reproducibility compared with previously reported datasets.

## 3 Data Description

### 3.1 RSM based performance evaluation and model validation

**Fig. 1:**
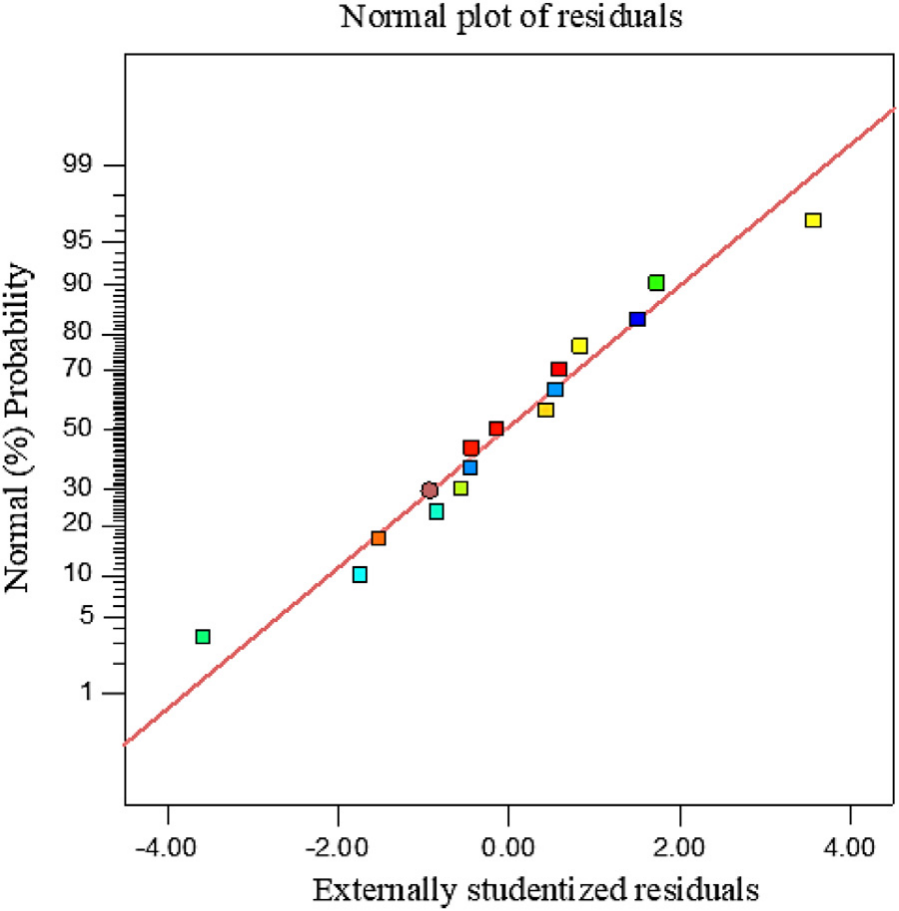
Normal probability plot of externally studentized residuals for evaluating the adequacy of the regression model.

The probability analysis of the externally studentized residuals, shown in Fig. 1, indicates that the residuals closely follow the reference straight line. Most of the data points are tightly clustered around the line, with only slight deviations observed at the extreme ends. The linear alignment of the residuals confirms that the fundamental assumption of normality for the regression model is satisfactorily met. In addition, the findings demonstrate that the experimental data are well captured by the optimized model, and the model predictions are statistically reliable. Therefore, the regression model used in this study is appropriate for describing the relationship between the input variables and the system response.

In this study, the performance of a photovoltaic (PV) solar-powered water pumping system installed in a coffee plantation area in Chiang Mai province was investigated using statistical analysis and the RSM. This method allows for the design of experiments involving three independent variables: solar radiation (W/m^2^), panel inclination (°), and panel surface temperature (°C). These parameters were used to analyze the performance of the solar-powered water pumping system for coffee cultivation. The experimental design considered the studied ranges of solar radiation (300–900 W/m2), panel inclination (15–35°), and panel surface temperature (30–60°C) as presented in Table 1. The performance results indicate that pump efficiency tends to increase with solar radiation and panel inclination, and that panel surface temperature directly affects the efficiency of the solar-powered water pump. Based on the experimental results, the pump efficiency ranged from 51.4% to 77.0%, with the highest efficiency of 77.0% obtained at a solar radiation of 600 W/m², a panel inclination of 25°, and a moderate panel surface temperature of 45°C. This condition represents the optimal operating point for coffee-growing areas. Eq. (1), which is the final equation based on actual factors, demonstrates that the system efficiency strongly depends on environmental and operating conditions. Eq. (1), expressed in terms of actual operating factors, is recommended for predictive modeling and practical application, whereas the coded-variable form was used primarily for statistical evaluation.

(1) $$\begin{array}{ccc} \mathrm{Pumping}\;\mathrm{efficiency}\;\left(\%\right) & = & -54.853+0.1549A+2.8956B+2.1723C-0.0003AB\\ & & -\;0.0002AC-0.0033BC-0.00010A^{2}-0.0532B^{2}-0.0255C^{2} \end{array}$$

**Table 1:** Pumping efficiency dataset of PV water pump under different environmental conditions.

Solar radiation (W/m^2^)	Panel inclination (°)	Panel surface temperature (°C)	Pumping efficiency (%)
900	35	45	68.3
900	25	60	65.1
600	15	60	61.6
600	25	45	76.3
600	15	30	70.6
300	15	45	55.2
900	15	45	72.0
900	25	30	74.5
600	35	60	59.4
300	25	60	51.4
600	25	45	77.0
600	25	45	76.5
300	35	45	54.9
600	35	30	70.5
300	25	30	57.8

Where: A = Solar irradiance, B = Panel inclination, C = Panel surface temperature.

**Fig. 2:**
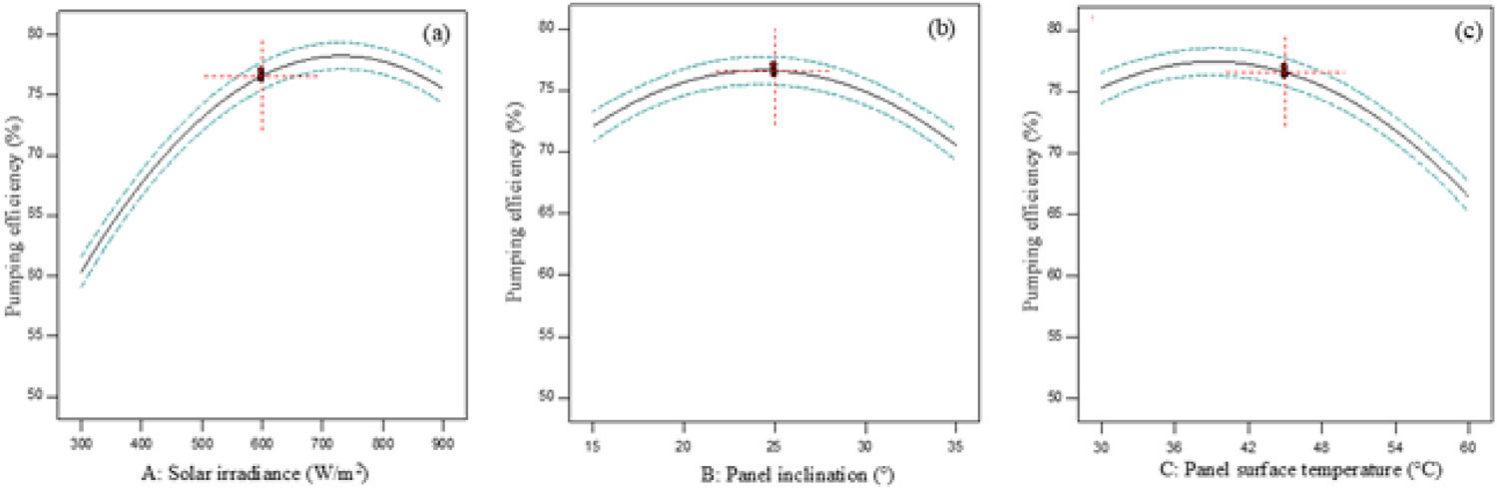
Perturbation plots illustrating the individual and interactive effects of solar irradiance, panel inclination, and panel surface temperature on the efficiency of the solar-powered water pumping system.

Meanwhile, further explanation is provided in Fig. 2, which presents the perturbation plots illustrating the individual effects of solar irradiance, panel inclination, and panel surface temperature on the pumping efficiency of the solar-powered water pumping system. The plots indicate that solar irradiance exerts the greatest influence on system efficiency, as evidenced by the steep curvature of the response near the optimal operating point. The pumping efficiency increases significantly as irradiance rises to approximately 600 W/m^2^. Beyond this point, the response begins to Y decline due to the nonlinear behavior of the system. Similarly, the panel inclination demonstrates a positive effect on the efficiency, with an optimal value of approximately 25°, at which the system achieves maximum light absorption. It is also observed that moderate heating at around 45°C slightly enhances pump efficiency, whereas excessively high temperatures reduce efficiency due to thermal losses in the photovoltaic cells.

From a reporting perspective [5] investigated the performance of a directly coupled photovoltaic water pumping system (PVWPS) by comparing the efficiency of the direct-coupled solar water pump with that of a conventional solar pumping system. The study revealed that optimizing the PVWPS design and incorporating electronic control systems significantly enhanced the system performance, particularly after applying the Maximum Power Point Tracking (MPPT) technique. Moreover, it was reported that the PVWPS is more cost-effective than diesel-powered pumps under various operating conditions, although the overall efficiency remained moderate (below 30%) due to the direct coupling configuration and environmental factors.

### 3.2 Statistical analysis of the response surface model

The statistical analysis of the response surface model using one-way analysis of variance (ANOVA) is presented in Table 2. Based on the statistical analysis equation, the results demonstrate the excellent adequacy of the RSM model, showing that the most significant factors in terms of solar irradiance and panel surface temperature exhibit p-values of less than 0.0001, including their interaction parameters. In addition, the model shows an R^2^ value of 0.9972, indicating that 99.72% of the variation in pump efficiency can be explained by the fitted quadratic model. The adjusted R^2^ (0.9922) and predicted R^2^ (0.9588) values are closely aligned, indicating that the model is reliable and exhibits strong predictive capability. Moreover, the low coefficient of variation (C.V. = 1.17%) and the high adequate precision ratio (40.72) confirm the accuracy and reproducibility of the experimental results. The analysis reveals that solar irradiance is the most influential variable affecting pump efficiency, followed by the panel and surface temperature. Increased solar irradiance enhances the energy conversion efficiency of the PV module, while an optimal panel inclination improves light absorption. In contrast, higher surface temperatures slightly reduce system efficiency due to heat losses within the photovoltaic cells.

**Table 2:** ANOVA for response surface quadratic model.

ANOVA for response surface quadratic model	List				
	SS	DF	MS	F-Value	p-value
Source					
Model	1072.9	9	119.2	199.1	>0.0001
A-Solar irradiance	462.1	1	462.1	771.9	>0.0001
B-Panel inclination	4.8	1	4.8	8.0	0.03693
C-Panel surface temperature	160.0	1	160.0	267.3	>0.0001
AB	2.7	1	2.7	4.6	0.08542
AC	2.1	1	2.1	3.5	0.11879
BC	1.0	1	1.0	1.7	0.25476
A^^2^	276.3	1	276.3	461.6	>0.0001
B^^2^	104.4	1	104.4	174.3	>0.0001
C^^2^	121.1	1	121.1	202.3	>0.0001

Among the independent factors, solar irradiance was identified as the most significant parameter (F = 771.92; p = 1.13 × 10^-6^), followed by panel surface temperature (F = 267.33; p = 1.56 × 10^-5^) and panel inclination (F = 7.98; p = 0.0369). The interaction terms (AB, AC, BC) were found to be statistically insignificant (p > 0.05), indicating that the combined effects of the variables exert less influence on the overall efficiency. The nonsignificant lack of fit (p = 0.127) confirms the adequacy and reliability of the model for predicting the system’s behavior within the studied range. Although the interaction terms are visualized in the response surface and contour plots to illustrate combined trends, their statistical contribution is relatively weak compared to the dominant main effects. Therefore, the system performance is primarily governed by the individual effects of solar irradiance and panel surface temperature rather than their interactions.

In addition, the regression coefficients were calculated and are presented in Table 3, describing the influence of each parameter among the different variables. The table shows that solar irradiance exhibits a positive coefficient (+7.6), indicating that increased sunlight directly enhances the pumping efficiency. In contrast, both the panel inclination (−0.7725) and panel surface temperature (−4.4725) have negative coefficients, reflecting a reduction in operational efficiency when the module temperature is high or the panel inclination is excessively large due to decreased energy conversion efficiency. Furthermore, the negative quadratic terms (A^2^, B^2^, C^2^) indicate that the efficiency does not increase linearly but instead reaches a peak before declining, which corresponds to the curvature observed in the response surface plots. Therefore, the model demonstrates that the optimal conditions occur at a solar irradiance of 600 W/m^2^, a panel inclination of 25°, and a surface temperature of 45°C, which correspond to the predicted maximum efficiency of 76.3–77.0%, demonstrating an effective improvement in the performance of the solar-powered water pumping system.

**Table 3:** RSM model coefficients and confidence intervals for PV water pump efficiency.

Factor	Coefficient		Standard	95% CI	95% CI
	Estimate	df	Error	Low	High
Intercept	76.60	1	0.447	75.452	77.748
A-Solar irradiance	7.60	1	0.274	6.897	8.303
B-Panel inclination	-0.77	1	0.274	-1.476	-0.069
C-panel surface temperature	-4.47	1	0.274	-5.176	-3.769
AB	-0.83	1	0.387	-1.822	0.167
AC	-0.73	1	0.387	-1.722	0.267
BC	-0.50	1	0.387	-1.492	0.497
A^2	-8.65	1	0.403	-9.686	-7.616
B^2	-5.32	1	0.403	-6.351	-4.281
C^2	-5.73	1	0.403	-6.761	-4.691

**Fig. 3:**
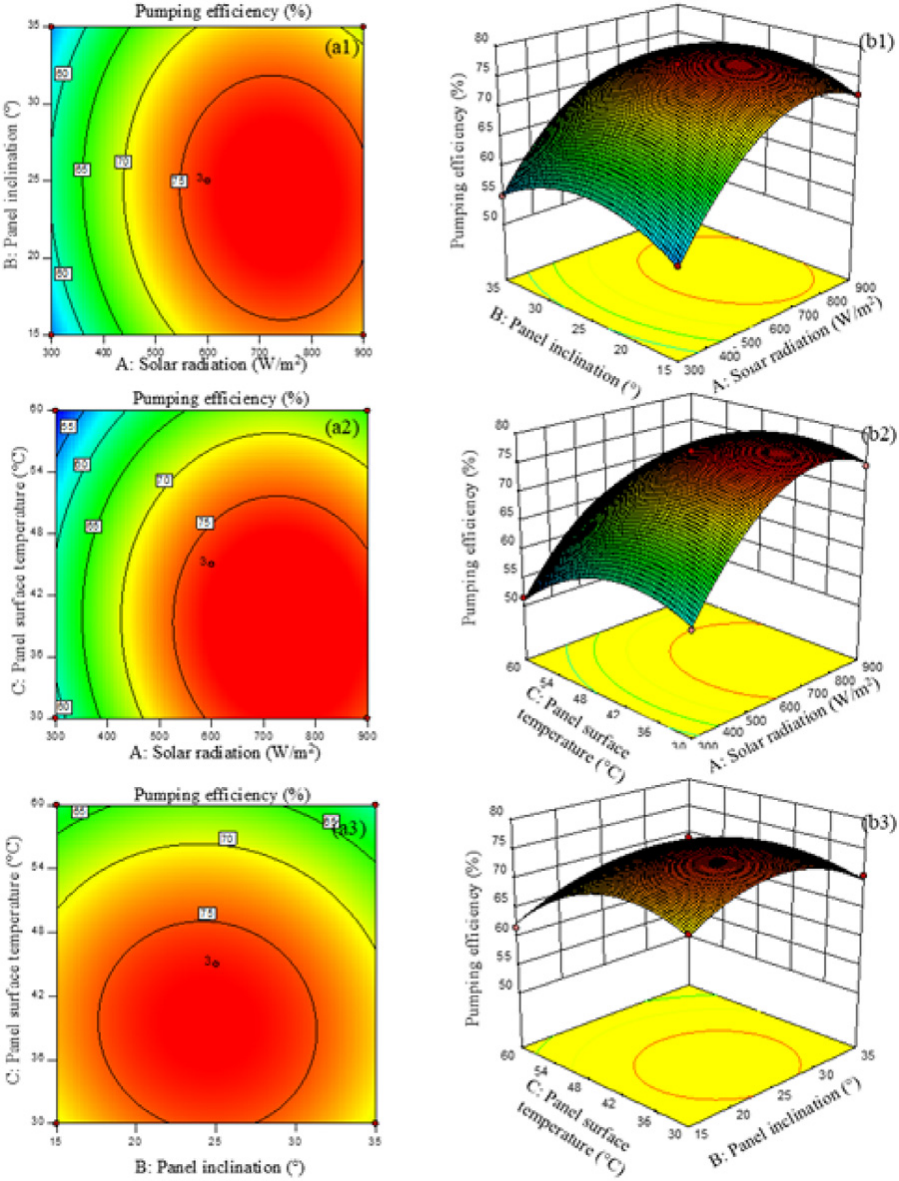
Response surface (a1-a3) and contour plots (b1-b3) showing the interactive effects of solar radiation, panel inclination, and panel surface temperature on pumping efficiency. Statistically significant differences were assessed using RSM ANOVA with a multiple comparison test, with significance values (∗p < 0.0001) reported.

In addition, Fig. 3 presents the 3D surface and contour plots illustrating the relationships among Solar radiation (W/m^2^), panel inclination (°), and panel surface temperature (°C) and their effects on the pumping efficiency of the solar-powered water pumping system. The contour and three-dimensional surface plots visualize the combined trends of the studied variables, while the statistical analysis confirms that the main effects dominate the system performance. The results show that the efficiency and graph profiles exhibit a downward-curved trend. In the first set of plots, the relationship between solar radiation and panel inclination (a1–b1) indicates that pumping efficiency increases with both solar irradiance and panel tilt angle, reaching its maximum at high irradiance levels and moderate tilt angles. This finding suggests that adjusting the panel to an optimal inclination improves solar light absorption. Meanwhile, Fig. 3 (a2–b2) shows that increasing solar irradiance enhances pumping efficiency; however, excessively high panel surface temperatures negatively affect performance due to thermal degradation of the electrical output of the photovoltaic cells, resulting in reduced efficiency. It is evident that effective heat dissipation can help maintain higher electrical output from the system.

At the same time, the relationship between panel inclination and panel surface temperature demonstrates that both parameters show combined trends in influencing efficiency, although their interaction effect is statistically limited. Therefore, as shown in a1–b1, this corresponds to the increased maximum efficiency of the solar-powered pump installed in Chiang Mai province, achieving the highest efficiency of 77.0% at a solar irradiance of 600 W/m^2^ under moderate panel inclination and panel surface temperature. The efficiency is more sensitive to solar irradiance and panel temperature than to inclination, and when conditions exceed the optimal range, performance decreases slightly, which may be attributed to thermal stress on the photovoltaic modules. Thus, RSM analysis effectively identifies the set of parameters that optimize system performance and serves as a visual tool for predicting efficiency under various environmental and operational conditions.

## 4 Experimental Design, Materials and Methods

### 4.1 Installation of the solar powered water pumping system

A solar-powered water pumping system was selected for use in this study*. The water pumping system* integrated with photovoltaic (PV) technology employs solar irradiance to generate electrical energy through the photovoltaic effect, which is subsequently used to drive the off-grid pump. It is necessary to investigate various parameters, including solar irradiance in the range of 300–900 W/m², panel inclination (15°–35°), and surface temperature (30–60°C), which are essential parameters for determining the performance of the solar-powered pumping system.

Solar irradiance was measured using a calibrated silicon-cell pyranometer (Apogee SP-110, accuracy ±5%), mounted in the same plane as the photovoltaic (PV) module to ensure representative irradiance measurements. The panel surface temperature was monitored using a contact-type temperature sensor (K-type thermocouple, accuracy ±1°C) attached to the rear surface of the PV panel. All measurements were recorded at fixed sampling intervals during system operation. Sensor calibration was conducted prior to data collection in accordance with the manufacturers’ specifications to ensure measurement accuracy and reproducibility.

### 4.2 Data analysis

Data analysis for response measurement was conducted using response surface methodology (RSM) implemented in design-expert software (version 10). A three-factor, three-level Box–Behnken design (BBD) was employed using design-expert software, requiring 15 experimental runs, including replicated center points, to estimate pure experimental error and evaluate model adequacy. This design is sufficient for fitting a full quadratic model while minimizing the number of experiments, with solar irradiance (300–900 W/m²), panel inclination (15°–35°), and panel surface temperature (30–60°C) coded at three levels (−1, 0, +1) to assess their individual and interactive effects on pump performance. Statistically significant effects were identified at p ≤ 0.01. The system response was modeled using a second-order polynomial equation incorporating linear, interaction, and quadratic terms, enabling quantitative assessment of the individual and combined effects of the operating parameters on pump performance (Eq. 2). The regression model was initially developed in terms of coded variables to evaluate the relative significance of the main and interaction effects through statistical analysis. For practical prediction and dataset reuse, the final regression equation was also expressed in terms of actual factors with physical units, which should be used for estimating pump efficiency under real operating conditions.

(2) $$\begin{array}{ccc} Y & = & \beta 0+\beta 1\times 1+\beta 2\times \;2+\beta 3\times \;3+\beta 12\times \;1\times \;2\\ & & +\;\beta \;13\times 1\times 3+\beta 23\times 2\times 3+\beta 11\times 21+\beta 22\times 22+\beta 33\times 23 \end{array}$$

Where Y is the predicted response; X1, X2, and X3 are the independent variables; β0 is a constant; β1, β2, and β3 are the linear coefficients; β12, β13, and β23 are the interaction coefficients; and β11, β22, and β33 are the quadratic coefficients. The fitted quadratic polynomial was utilized to obtain 3D surface plots of the correlations between the independent variables and response

## Limitations

Not applicable.

## Ethics Statement

The work did not involve the use of human subjects, animal experiments and data collected from social media platforms.

## CRediT Author Statement

**Nopparat Suriyachai**: Writing – original draft, Writing – review & editing, Methodology, Investigation, Data curation; **Torpong Kreetachat**: Data curation, Formal analysis, Validation, Investigation; **Saksit Imman**: Writing – original draft, Writing – review & editing, Funding acquisition, Supervision, Conceptualization, Methodology, Validation, Resources.

## Data Availability

Mendeley DataDataset on the Performance of a Photovoltaic Solar Water Pump in Coffee Plantations Using Response Surface Methodology (RSM) (Original data).
